# Dissecting Tumor Antigens and Immune Subtypes of Glioma to Develop mRNA Vaccine

**DOI:** 10.3389/fimmu.2021.709986

**Published:** 2021-08-27

**Authors:** Hua Zhong, Shuai Liu, Fang Cao, Yi Zhao, Jianguo Zhou, Feng Tang, Zhaohua Peng, Yangsheng Li, Shen Xu, Chunlin Wang, Guohua Yang, Zhi-Qiang Li

**Affiliations:** ^1^College of Life Sciences, Wuhan University, Wuhan, China; ^2^Department of Biochemistry, Molecular Biology, Entomology and Plant Pathology, Mississippi State University, Starkville, MS, United States; ^3^Department of Cerebrovascular Disease, Affiliated Hospital of Zunyi Medical University, Zunyi, China; ^4^Department of Medical Genetics, School of Basic Medical Science, Demonstration Center for Experimental Basic Medicine Education, Wuhan University, Wuhan, China; ^5^Department of Oncology, The Second Affiliated Hospital of Zunyi Medical University, Zunyi, China; ^6^Department of Radiation Oncology, Universitätsklinikum Erlangen, Erlangen, Germany; ^7^Department of Neurosurgery, Zhongnan Hospital, Wuhan University, Wuhan, China; ^8^Department of Neurosurgery, No. 901 Hospital of the Chinese People’s Liberation Army Logistic Support Force, Hefei, China

**Keywords:** glioma, mRNA vaccine, tumor antigens, immune subtypes, immune landscape

## Abstract

**Background:**

Nowadays, researchers are leveraging the mRNA-based vaccine technology used to develop personalized immunotherapy for cancer. However, its application against glioma is still in its infancy. In this study, the applicable candidates were excavated for mRNA vaccine treatment in the perspective of immune regulation, and suitable glioma recipients with corresponding immune subtypes were further investigated.

**Methods:**

The RNA-seq data and clinical information of 702 and 325 patients were recruited from TCGA and CGGA, separately. The genetic alteration profile was visualized and compared by cBioPortal. Then, we explored prognostic outcomes and immune correlations of the selected antigens to validate their clinical relevance. The prognostic index was measured *via* GEPIA2, and infiltration of antigen-presenting cells (APCs) was calculated and visualized by TIMER. Based on immune-related gene expression, immune subtypes of glioma were identified using consensus clustering analysis. Moreover, the immune landscape was visualized by graph learning-based dimensionality reduction analysis.

**Results:**

Four glioma antigens, namely ANXA5, FKBP10, MSN, and PYGL, associated with superior prognoses and infiltration of APCs were selected. Three immune subtypes IS1–IS3 were identified, which fundamentally differed in molecular, cellular, and clinical signatures. Patients in subtypes IS2 and IS3 carried immunologically cold phenotypes, whereas those in IS1 carried immunologically hot phenotype. Particularly, patients in subtypes IS3 and IS2 demonstrated better outcomes than that in IS1. Expression profiles of immune checkpoints and immunogenic cell death (ICD) modulators showed a difference among IS1–IS3 tumors. Ultimately, the immune landscape of glioma elucidated considerable heterogeneity not only between individual patients but also within the same immune subtype.

**Conclusions:**

ANXA5, FKBP10, MSN, and PYGL are identified as potential antigens for anti-glioma mRNA vaccine production, specifically for patients in immune subtypes 2 and 3. In summary, this study may shed new light on the promising approaches of immunotherapy, such as devising mRNA vaccination tailored to applicable glioma recipients.

## Background

As the most common primary intracranial tumor in adults, glioma represents 81% of malignant brain tumors and causes high morbidity and mortality (1). Currently, the conventional treatment for glioma includes surgical removal of the tumor followed by chemotherapy and radiotherapy (2). However, the clinical outcomes of patients are still very poor, with serious side effects, a high risk of resistance, and a median overall survival time of only 15 months (3, 4). Hence, it is urgent and necessary to find novel and patient-specific strategies to improve the therapeutic condition of glioma.

Since surviving several malignancies successfully, tumor immunotherapeutic approaches have been paid more attention by oncologists. Among them, preventive and therapeutic vaccines against tumors are full of potential and attractive. These vaccines can specifically attack and destroy malignant tumor cells that express tumor-associated antigens or tumor-specific antigens and achieve chronic therapeutic effects based on immune memory (5). Cancer vaccines fall mainly into four categories, tumor cell, dendritic cell, DNA, and RNA types based on the antigen form (6, 7). Compared with conventional approaches, mRNA vaccine has its major advantages (8, 9): (1) Safety profile: mRNA vaccine is non-infectious,which means it is not made with pathogen particles or inactivated pathogen. And mRNA does not integrate itself into the host genome or excluded irrelevantly. Once the protein is made, mRNA is degraded by cellular RNases with a short and regulatable half-life *in vivo*. (2) High efficacy: In the context of clinical trial results, the mRNA vaccine is well tolerated by healthy individuals, with few side effects after eliciting a reliable immune response. Plus, mRNA sequences can be easily designed to encode any pathological antigen, which is conducive to individualized therapies. (3) Easily manufactured: mRNA vaccines can be produced more rapidly under standardized processes improving responsiveness to emerging outbreaks.

To date, over 50 clinical trials for RNA vaccines were performed against blood cancers, melanoma, glioblastoma, and prostate cancer (https://clinicaltrials.gov/). Due to tumor heterogeneity and complex immune microenvironment, the application of effective antiglioma mRNA vaccine remains largely uncharacterized, and it will require a better understanding of suitable patient subpopulation for vaccination.

In the present study, we aimed to identify potential antigens of glioma for the development of antiglioma mRNA vaccines. Four glioma antigens involved in superior prognoses and infiltration of antigen-presenting cells were defined. Then, we determined immune subtypes of glioma for the selection of suitable recipients from an extremely heterogeneous population. Defined three immune subtypes varied in cellular, molecular, and clinical features, which were consistent in CGGA and TCGA repositories. Our findings might provide new insight into tumor immunotherapy and provide a valuable reference for cancer vaccine development.

## Methods

### Data Extraction and Preprocessing

Normalized RNA-seq and survival information of 325 glioma patients were employed from the Chinese Glioma Genome Atlas (CGGA, https://www.CGGA-argo.org) (10). Those of 173 GBM (glioblastoma) patients and 529 LGG (low-grade glioma) patients from The Cancer Genome Atlas (TCGA, https://www.cancer.gov/tcga) were downloaded from UCSC Xena (http://xena.ucsc.edu/). Firstly, we screened tumor samples and excluded tumor samples that lacked clinical data. Then, the FPKM values were translated to log_2_(FPKM+1). The batch effect before merging different expression matrixes was removed using the “ComBat” function in the “SVA” R package. A list of 4,815 genes related to immunity was extracted from The Immunology Database and Analysis Portal (ImmPort, https://www.immport.org/home), which was automatically generated by searching EntrezGene and Gene Ontology (GO) terms using keywords related to immunology. Then, immunologists checked various sources of literature and compiled the list manually. Finally, 3,576 immunologically related genes in the CGGA and TCGA cohorts were used in subsequent analysis.

### Differential Gene Expression, Mutation, and Survival Analysis

We integrated the RNA-seq data of TCGA using the cBioPortal for Cancer Genomics (http://www.cbioportal.org) (11, 12) and compared the genetic variation of glioma. Through the Gene Expression Profiling Interactive Analysis (GEPIA2, http://gepia2.cancer-pku.cn) (13), we integrated the differential gene expression and patient survival data from the TCGA cohort. The genes identified by ANOVA with log_2_ fold change > 3 and q value < 0.05 were considered to be significantly upregulated. Based on the Kaplan–Meier method with a 50% (Median) cutoff, overall survival (OS) and disease-free survival (DFS) were calculated and then compared by the log-rank test. The hazards ratio was evaluated *via* the Cox proportional hazards regression model. The prognostic index of immune subtypes was assessed using the “survival” and “survminer” R packages.

### Identification and Validation of the Immune Subtypes

Based on the combined expression profile of CGGA and TCGA, 3,576 immune-related genes were gathered, and consensus clustering was applied to identify a robust cluster of 1,027 glioma patients (14). The 1,000 bootstraps with 80% item resampling and a range of K from 2 to 10 were selected for clustering analysis. Partition around the medoids classifier was trained in the discovery cohort. By calculating the in-group proportion and Euclidean correlation in the centroid of gene module scores, we quantitatively acquired and verified the consistency of immune subtypes among populations.

### Immune Cell Infiltration Estimation With TIMER and ssGSEA

We used the Tumor Immune Estimation Resource (TIMER, https://cistrome.shinyapps.io/timer/) (15) to analyze and visualize the Spearman correlation between glioma-related genes and numerous tumor-infiltrating immune cells (TIICs). Statistical significance was determined by p-value < 0.05. The single-sample gene set enrichment analysis (ssGSEA) quantified the relative infiltration over 24 TIICs in the glioma microenvironment (16). The normalized expression matrix was compared with the published gene set for predicting the abundance of 24 TIICs using the “GSVA” R package (17). Then, the relative abundance enrichment score of each immune cell type was measured and standardized from 0 to 1.

To characterize the immune status and antigenome of tumors, immunophenoscore (IPS) was evaluated referring to the previous study (18), which was created based on the gene expression comprising four immune categories: suppressor cells, effector cells, major histocompatibility complex (MHC) molecules, and immunomodulators.

### Construction of Immune Landscape

Based on the immune gene expression profile, the graph learning-based dimensionality reduction analysis was implemented using discriminative dimensionality reduction with tree (DDRTree) to uncover the distribution of immune subtypes of an individual patient. The immune landscape was visualized in the plot cell trajectory function by the “monocle” R package.

To further uncover the intrinsic structure and distribution of individual patients, we extended a graph learning-based dimensionality reduction analysis to the immune gene expression profiles. The discriminative dimensionality reduction with trees (DDRTree) was used, and the immune landscape was visualized with the plot cell trajectory function (package monocle) (19).

### Weighted Gene Coexpression Network Analysis

The coexpression modules of immune-related genes was recognized using the “WGCNA” R package (20). To develop a weighted adjacency matrix, the soft threshold power of β was set to β=6 using the scale-free topology criterion. The bottom-up algorithm and dynamic tree cut method was chosen to recognize coexpression modules, and module eigengenes (MEs) were estimated to quantify module similarity. The Z-summary was calculated to estimate the conserved modules with default settings and 200 permutations. Then, GO (Gene Ontology) and KEGG (Kyoto Encyclopedia of Genes and Genomes) enrichment analyses of genes in the module were used to explore gene functions and pathways related to immune-related molecules and cell characteristics through the “clusterProfiler” R package (21). Benjamini–Hochberg adjusted p-value less than 0.05 was taken to present statistical significance.

## Results

### Candidate Antigens of Glioma Detection

**Figure 1:**
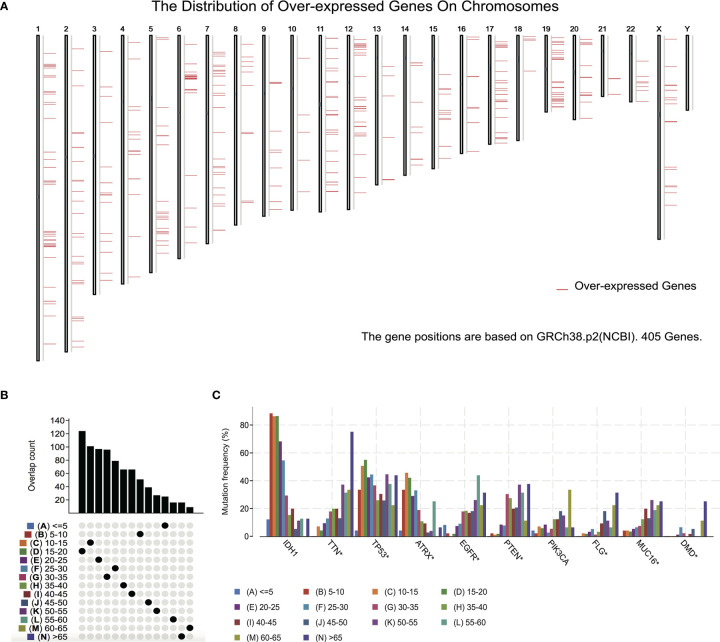
Identification of potential tumor antigens of GBM. **(A)** Identification of potential tumor-associated antigens of glioma. Chromosomal distribution of overexpressed genes in glioma as indicated. **(B, C)** Identification of potential tumor-specific antigens of glioma. **(B)** Samples overlapping in mutation count groups. **(C)** Genes with the highest frequency in mutation count groups.

**Figure 2:**
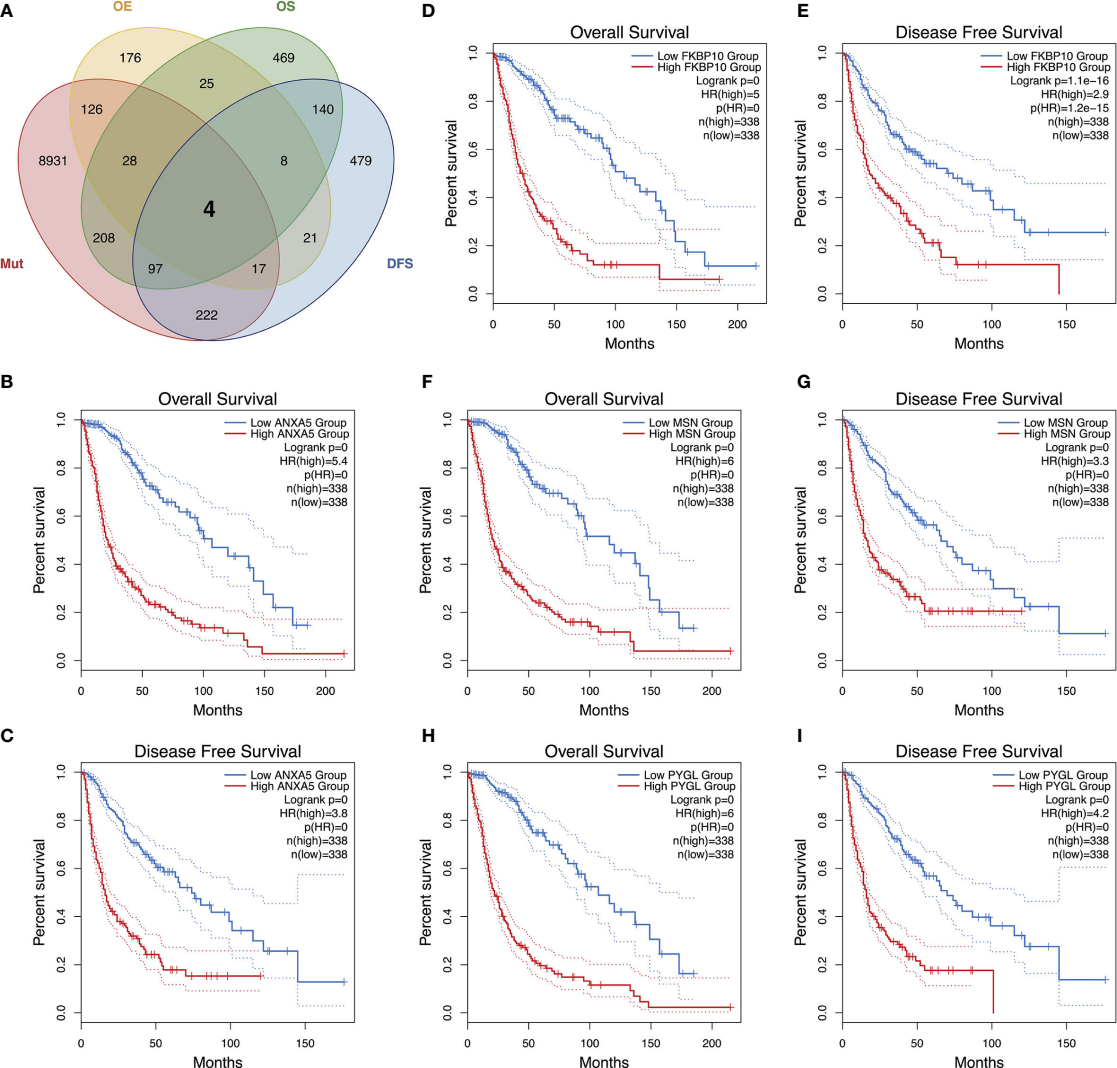
Identification of tumor antigens associated with glioma prognosis. An upset plot of potential tumor antigen candidates with overexpression (OE), mutation (Mut), OS, and DFS in glioma **(A)**. Kaplan–Meier OS and DFS curves comparing the groups with high and low expressions of ANXA5 **(B, C)**, FKBP10 **(D, E)**, MSN **(F, G)**, and PYGL **(H, I)** in glioma.

**Figure 3:**
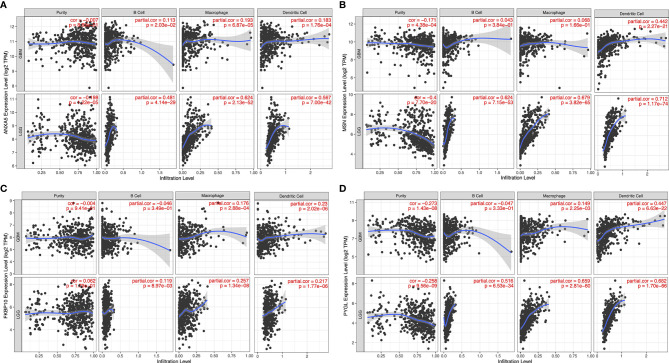
Identification of tumor antigens associated with APCs. Correlation between the expression levels of ANXA5 **(A)**, MSN **(B)**, FKBP10 **(C)**, and PYGL **(D)** and infiltration of macrophages, dendritic cells, and B cells in GBM and LGG tumors.

To detect candidate antigens of glioma, the aberrantly expressed genes of glioma were screened at first. A total of 797 dysregulated genes were detected. Among them, 405 elevated expressed genes were of interest as coding tumor-associated antigens (**Figure 1A**). Then, 9,632 mutated genes potentially encoding for tumor-specific antigens were identified after analyzing mutation counts in individual samples. As shown in **Figure 1B**, glioma was featured by low immunogenicity, since low mutation count (15–20) accounted for the highest proportion. Top 10 high mutation counts were displayed in IDH1, TTN, TP53, ATRX, EGFR, PTEN, PIK3CA, FLG, MUC16, and DMD (**Figure 1C**). Eventually, 175 upregulated and mutated tumor-specific genes were filtered. To narrow down the potential genes for developing mRNA vaccine against glioma, those who serve to forecast the prognosis of glioma were further selected. Thirty-two genes were predominantly associated with the overall survival (OS) of glioma patients, and four disease-free survival (DFS)-related genes were identified conclusively (**Figure 2A**). The amplified expressions of ANXA5 (**Figures 2B, C**), FKBP10 (**Figures 2D, E**), MSN (**Figures 2F, G**), and PYGL (**Figures 2H, I**) were correlated with favorable prognosis OS and DFS of glioma, which exert pivotal roles in glioma development and progression. Furthermore, we found that all high expression levels of ANXA5, FKBP10, MSN, and PYGL showed significant correlation with the OS in WHO III patients, whereas they had no significant correlation with WHO IV (**Figure S1**). As for immune cell infiltration estimation analysis, elevated expressions of ANXA5 and MSN were exhibited in increased infiltration of B cells, macrophages, and DCs (**Figures 3A, B**). Meanwhile, FKBP10 and PYGL displayed a similar tendency in enhanced infiltration of immune cells with some fluctuant (**Figures 3C, D**). Thus, ANXA5, FKBP10, MSN, and PYGL might be presented by APCs to the T cells and recognized by the B cells to induce a tumor response. Taken together, four promising candidates were identified as tumor antigens for developing glioma-mRNA vaccines.

### Potential Immune Subtypes of Glioma Identification

**Figure 4:**
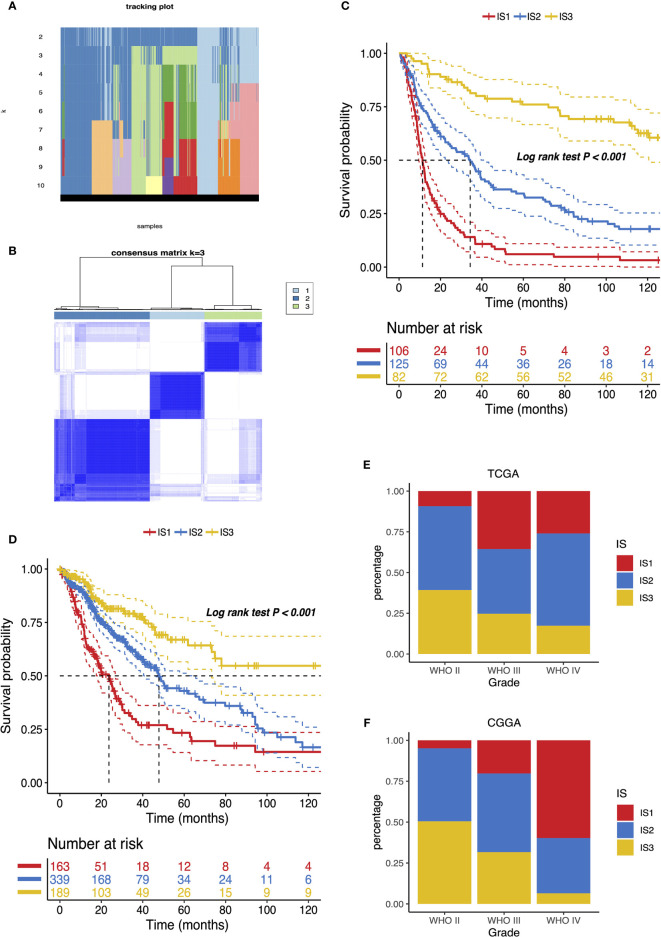
Identification of potential immune subtypes of glioma. **(A)** Tracking plot of k in the consensus cluster of immune-related genes. **(B)** Sample clustering heat map. Kaplan–Meier curves showing OS of glioma immune subtypes in CGGA **(C)** and TCGA **(D)** cohorts. Distribution of IS1–IS3 across glioma WHO grades II to IV in CGGA **(E)** and TCGA **(F)** cohorts.

We next constructed immunotypes to reflect tumor immune status and their microenvironments. An expression profile of 3,576 common immune-related genes in 1,027 patients from both CGGA and TCGA databases was used to implement the consensus clustering. Based on the tracking plot, k = 3 was set resulting in defining three distinct immune subtypes (IS) as IS1, IS2, and IS3 (**Figures 4A, B**). We found that IS3 glioma carried the best outcome, whereas IS1 glioma showed the poorest survival probability out of the three subtypes in both CGGA and TCGA cohorts (**Figures 4C, D**). The association of four antigens ANXA5, FKBP10, MSN, and PYGL with IS1–IS3 subtype prognosis was also explored. As shown in **Figure S2**, patients overexpressing ANXA5, FKBP10, MSN, and PYGL in the tumor tissues had significantly shorter OS compared to the low expressed group in IS1–IS3 subtypes. Overall, the OS prognosis of IS1 was worse than IS2 and IS3 subtypes for four antigens ANXA5, FKBP10, MSN, and PYGL.

The distribution of the immune subtype across different WHO grades manifested that patients diagnosed as differential grades were clustered unevenly, while IS2 and IS3 accounted for the major proportion of WHO II (**Figure 4E**). On the other hand, LGG (WHO II and III grade) was in the majority in IS2 and IS3 (**Figure S3**). In accord with the results analyzed by the CGGA cohort, the immune subtypes were significantly altered among distinct grades in the TCGA database, where IS3 was predominantly associated with WHO grade II. However, a substantial correlation existed between WHO IV and IS1 (**Figures 4F** and **S3**). Since a previous study reported that decreasing survival is correlated with increasing grade (22), these observations explained part of the prognosis difference of three immune subtypes. Above all, the immunotyping provides an indication of predicting the prognosis of glioma patients whose accuracy beats traditional grading.

### Association Between Immune Subtypes and Mutational Status

**Figure 5:**
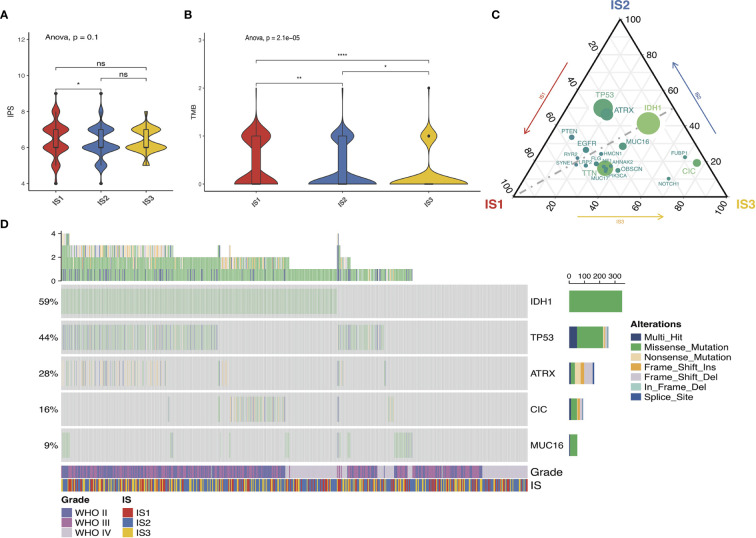
Association between immune subtypes and mutation. IPS and TMB in IS1–IS3 across TCGA **(A, B)**. Distribution of top 20 mutated genes among three immune subtypes in the TCGA **(C)** database. Seven highly mutated genes in glioma immune subtypes in TCGA **(D)** dataset. ns presents no difference, *p < 0.05, **p < 0.01, and ****p < 0.0001.

The immunophenotypic score (IPS) can be used to determine the tumor immunogenicity (18, 23). Herein, we utilized the IPS to determine the association between immune subtypes and immune response. Additionally, the tumor mutational burden (TMB) was assessed for the immune three subtypes in view of its close relationship with immunotherapeutic efficacy (24, 25). The mutations in each patient were calculated using the TCGA mutect2-processed mutation data. As shown in **Figure 5A**, no significant difference existed among the three subtypes. Similar trends were obtained in the TMB as well (**Figure 5B**). Twenty genes including IDH1, TP53, and ATRX were most frequently altered in each subtype across the TCGA cohort (**Figure 5C**). Then, the landscape of seven common immune-related genes with the most frequent genomic mutation was also delineated (**Figure 5D**). The results indicated that the quantities of tumor antigens encoded by mutated genes differed insignificantly among the three immune subtypes. In particular, highest mutation counts of antigen ANXA5 and PYGL were observed in IS3 and IS2, separately (**Table S1**).

### The Association of Immune Modulators With Immune Subtypes of Glioma

**Figure 6:**
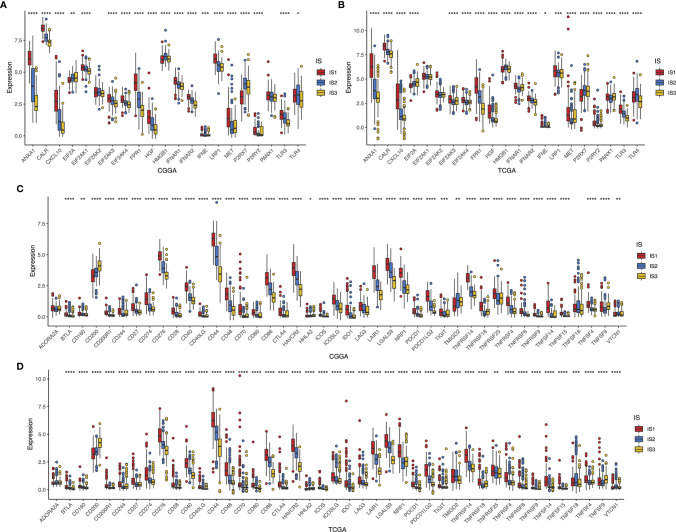
Association between immune subtypes and ICPs and ICD modulators. Differential expression of ICD modulator genes among the glioma immune subtypes in CGGA **(A)** and TCGA **(B)** cohorts. Differential expression of ICP genes among the glioma immune subtypes in CGGA **(C)** and TCGA **(D)** cohorts. *p < 0.05, **p < 0.01, ***p < 0.001, and ****p < 0.0001.

Considering the importance of immunogenic cell death (ICD) modulators and immune checkpoints (ICPs) in modulating host antitumor immunity, their expression levels in the different subtypes were measured (26). The overall expression patterns of ICDs and ICPs in the CGGA cohort were similar to those in the TCGA cohort. A total of 41 ICDs were detected, of which 19 (90.48%) genes in both CGGA (**Figure 6A**) and TCGA datasets (**Figure 6B**) showed significant differences among the immune subtypes, respectively. Among them, EIF2A, HMGB1, IFNE, P2RX7, and TLR4 were predominantly enriched in IS2 or IS3 tumors simultaneously in CGGA and TCGA databases. On the other hand, 43 ICPs-associated genes were extracted in both cohorts, of which 41 (95.34%) genes in the CGGA (**Figure 6C**) and 42 (97.67%) in the TCGA databases (**Figure 6D**) were differentially expressed among IS1–3. For instance, IS1 showed significant upregulation of CD244, CD27, CD274, CD276, CD28, CD40, CD44, CD48, CD70, CD80, CTLA4, HAVCR2, ICOSLG, IDO1, LAG3, LAIR1, LGals9, NRP1, PDCD1LG2, TNFRSF14, TNFRSF18, TNFRSF4, TNFSF14, and TNFSF4 across the two databases, while these genes were down-expressed separately in the IS2 and IS3 tumors. Overall, immunotyping can mirror the expression profiles of ICD modulators and ICPs, acting as promising therapeutic biomarkers for mRNA vaccination.

### Cellular and Molecular Features of Immune Subtypes

**Figure 7:**
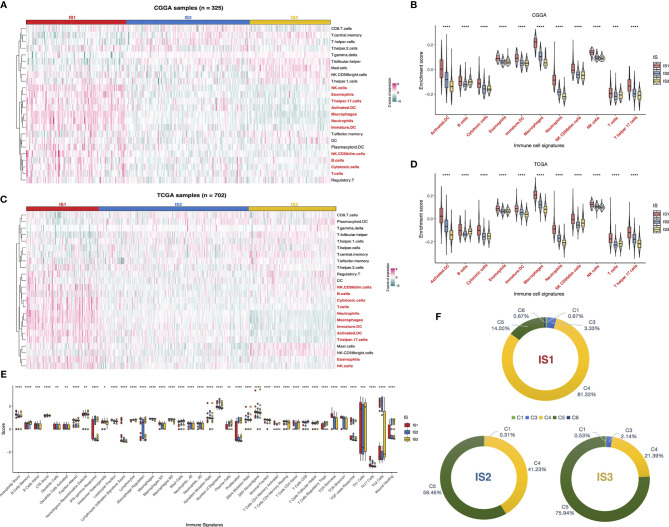
Cellular and molecular characteristics of immune subtypes. Differential enrichment scores of 24 immune cell signatures among glioma immune subtypes in CGGA **(A)** and TCGA **(C)** cohorts. Differential enrichment scores of 11 immune cell signatures enriched in IS1 across CGGA **(B)** and TCGA **(D)** cohorts. Differential enrichment scores of 56 immune signatures among glioma immune subtypes and 40 significant immune signatures with p-value<0.05 **(E)**. Overlap of three immune subtypes with six pan-cancer immune subtypes in glioma **(F)**. *p < 0.05, **p < 0.01, ***p < 0.001, and ****p < 0.0001.

Considering that tumor immune status influences the response to the mRNA vaccine, the immune cell components in IS1–3 were next characterized. We scored 24 past reported signature genes by ssGSEA across TCGA and CGGA cohorts (16). The immune cell components were stratified into three categories and displayed high diversity among the subtypes (**Figure 7A**). Of those, the enrichment scores of natural killer (NK) cells, eosinophils, T helper 17 cells, activated DC, macrophages, neutrophils, immature DC, NK CD56^dim^ cells, B cells, cytotoxic cells, and T cells were highly accumulated in IS1 compared to IS2 and IS3 (**Figure 7B**). Thus, IS1 was regarded as immunologically “hot,” while IS2 and IS3 were termed immunologically “cold.” Subsequent analyses in the TCGA cohort have come up with similar results (**Figures 7C, D**). These findings illuminated that the immunotyping reflects the glioma immune status in an attempt to investigate suitable recipients for mRNA vaccination. The immune infiltration in patients with immunologically desert IS2 and IS3 could be triggered by mRNA vaccine with these antigens.

Besides, the correlation between the immune subtypes and 56 previously reported molecular signatures (27) was evaluated to validate the reliability of the immune subtype. Finally, 40 immune-associated signatures were filtered as statistically significant with a p-value < 0.05 as the threshold (**Figure 7E**). Of these, IS1 was dominated by leukocyte fraction, TCR richness, macrophage regulation, and stromal fraction, which pointed to an immunosuppressive phenotype. In contrast, the low scores of those cellular signatures in IS2 and IS3 were indicative of immunologically cold phenotypes.

We exploited sequentially the relationship between the three immune subtypes and previously defined C1–C6 pan-cancer immune subtypes, of which glioma was mostly divided into C1, C3, C4, C5, and C6 (27). A distribution difference over the C1–C6 proportion in IS1–3 was observed. C4 (lymphocyte depleted) was prevalent in IS1 (81.33%), as C5 (immunologically quiet) was highly clustered into IS2 (58.46%) and IS3 (75.94%). On the other hand, only C1, C4, and C5 existed in IS2, while IS1 contained C6 exclusively (**Figure 7F**). Thus, the above findings not only demonstrated the unique features of the glioma immune microenvironment but also provided a conducive complement to past reports. In summary, the immune subtypes signaled the cellular and molecular characteristics in glioma patients, distinguishing potentially appropriate targets for mRNA vaccination.

### The Immune Landscape of Glioma

**Figure 8:**
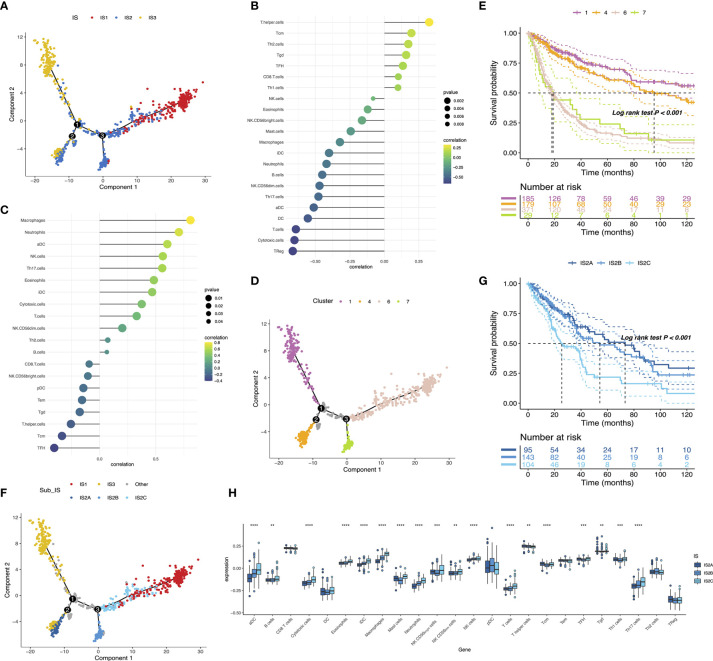
The immune landscape of glioma **(A)**. Each point represents a patient and the immune subtypes are color-coded. Significant correlation of PC1 **(B)** and PC2 **(C)** with immune cell signatures. The immune landscape of samples from four extreme locations **(D)** and their prognoses **(E)**. The immune landscape of the subsets of glioma immune subtypes **(F)**. Different groups in IS2 are associated with different prognostic statuses **(G)**. Differential enrichment scores of 24 immune cell signatures in the IS2A-C subsets **(H)**. **p < 0.01, ***p < 0.0001, and ****p < 0.0001.

The immune landscape of glioma was established using a reservoir of immune-related gene expression profiles (**Figure 8A**). In **Figures 8B, C**, the first principal component (PC1) was positively linked with various immune cells, such as CD8 T cells, T helper cells, TFH, Tcm, Th2 cells, Th1 cells, and Tgd, while immature DC showed a positive association with the second principal component (PC2). In addition, extremely distributed samples in the immune landscape underwent prognostic analysis. We found that clusters 1 and 4 had superior survival probability than clusters 6 and 7. The observations combined with the aforementioned results suggested that an immune landscape based on immune subtypes could be developed for patient prognosis prediction (**Figures 8D, E**). Remarkably, the integral location of one subtype was opposite to that of the others. The opposing distribution was even displayed within subtypes especially in IS2. According to the distribution of immune cell populations, IS2 was further divided into IS2A, IS2B, and IS2C (**Figure 8F**). Follow-up prognosis analysis showed that patients with IS2A referred to the best survival among those with three subsets (**Figure 8G**). The enrichment scores of certain immune cells were also considerably varied. IS2A scored lower in terms of B cells, activated DC, T cells, and Th17 cells (**Figure 8H**). These outcomes revealed significant intracluster heterogeneity within immune subtypes. In summary, the immune landscape of glioma based on immune subtypes could precisely determine the immune components of each patient and predict their prognoses, facilitating the individualized treatment for mRNA vaccine.

### Detection of Immune Gene Coexpression Modules of Glioma

**Figure 9:**
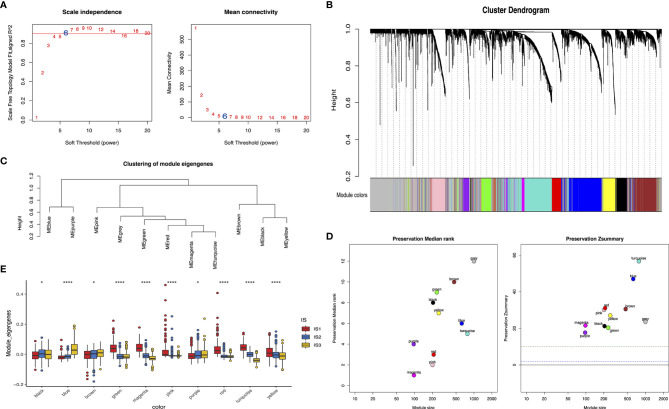
Identification of immune gene coexpression modules of glioma. Scale-free fit index for various soft-thresholding powers (β) and mean connectivity for various soft-thresholding powers **(A)**. Dendrogram of all differentially expressed genes clustered based on a dissimilarity measure (1-TOM) **(B)**. Module eigengenes clustering tree **(C)**. Module preservation statistics of gene modules. The x-axis presents the module size as the y-axis presents the preservation median rank or preservation Z-summary **(D)**. Differential distribution of MEs of each module in glioma subtypes **(E)**. *p < 0.05 and ****p < 0.0001.

**Figure 10:**
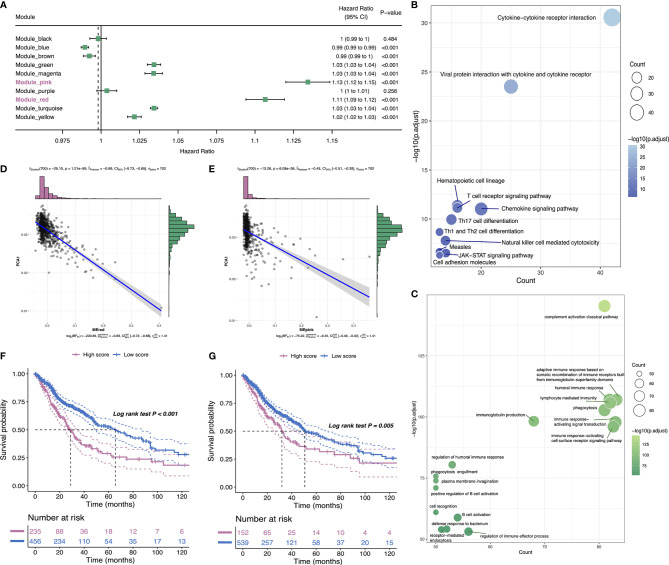
Identification of immune hub genes of glioma. Forest maps of single factor survival analysis of 10 modules of glioma **(A)**. Bubble plots showing top KEGG terms enriched in the red **(B)** and top GO terms enriched in pink **(C)** modules. The dot size and color intensity represent the gene count and -log10 (adjusted p-value), respectively. Correlation between PC1 and red **(D)** and pink **(E)** module feature vector. Differential prognosis in red **(F)** and pink **(G)** modules with high and low mean.

The weighted gene coexpression network analysis (WGCNA) was performed to detect immune gene coexpression modules, in which densely interconnected genes clustered. The soft threshold was chosen at β = 6 for a scale-free network, while scale-free topology model-fit R^2^ was equal to 0.87 (**Figure 9A**). The module colors were represented by the degree of dissimilarity after building a hierarchy clustering dendrogram (**Figure 9B**). The module eigengenes (MEs) were calculated, and similar modules were merged with height = 0.25 and minimum module size = 30 (**Figure 9C**). The genes not clustered into the rest were assigned to the gray module. Eventually, 10 coexpression preserved modules (Z-summary score > 10, except the gray module) were obtained with module size ranging from 97 genes in the magenta module to 776 genes in the turquoise module (**Figure 9D**). The MEs of 10 modules in three immune subtypes were further analyzed (**Figure 9E**). IS3 had the lowest MEs in green, magenta, pink, red, yellow, and turquoise modules, whereas IS1 showed the highest eigengenes in these modules correspondingly. Thereby, IS3 corresponded to immunologically cold and IS1 to inflamed tumors. The prognostic correlation results indicated that the red and pink modules were closely correlated with the prognosis of glioma (**Figure 10A**). Furthermore, functional enrichment terms elucidated genes in the red module were involved in cytokine–cytokine receptor interaction, Th17 cell differentiation, and JAK-STAT signaling pathway (**Figure 10B**), which had evidently negative association with PC1 in the immune landscape (**Figure 10D**). The genes in the pink module were enriched in the complement activation classical pathway, lymphocyte mediated immunity, and phagocytosis (**Figure 10C**), showing an apparently negative correlation with PC1 in the immune landscape (**Figure 10E**). Consistent with the abovementioned findings, patients with lower scores of genes had prolonged survival time compared to those with higher scores in red (**Figure 10F**) and pink (**Figure 10G**) modules. Hence, the mRNA vaccine could be effective for patients who have high expression of genes categorized into red and pink modules. Ultimately, 12 and 13 hub genes with relevance > 90% to MEs of red and pink modules were identified, respectively, such as CD3G and IGLL5, which are potential biomarkers for mRNA vaccine. Therefore, these hub genes can exert as prognostic and predictive biomarkers to identify suitable glioma patients for mRNA vaccine application.

## Discussion

As a hotspot in cancer immunotherapy after emerging the technological breakthroughs, the mRNA vaccine encoding specific antigen has high safety, has excellent therapeutic effect, and is cost-effective and can be produced on a large scale with promising outcomes in treatment and prevention (28). Nonetheless, only a fraction of people who received cancer immunotherapy respond favorably. One of the feasible solutions is to administer a cancer vaccine that encodes for peptides that contain mutations found in the tumor (29). In this study, we work to find neoantigens unique to glioma that contribute to creating the personalized mRNA-based vaccine. The differentially expressed and mutational profile of glioma was constructed, and four targetable antigens (ANXA5, FKBP10, MSN, and PYGL) were further confirmed. Their overexpressions were associated with poor OS and DFS as well as high APCs and B cell infiltration. The findings revealed that these candidates have an outsize impact on the progression and prognosis of glioma. Although these antigens need more in-depth clinical evaluation, their potential for glioma-related mRNA development has been consolidated by previous studies. For example, Moesin (MSN) activates tumor progression by interacting with CD44 to induce Wnt/b-catenin pathway proliferation. It suggests that MSN can be used as a marker for glioma progression and a drug target (30). Studies have reported that FKBP10 participates in the proliferation of glioma cells *via* interacting with Hsp47 and activating the AKT-CREB-PCNA axis. Inhibiting tumor progression by inhibiting FKBP10-related signals may provide a potential treatment option for glioma (31). As for PYGL, it encodes glycogen phosphorylase in cells and is related to cell metabolism. PYGL is proposed as a hypoxia signal with prognostic significance in head and neck squamous cell carcinoma and breast cancer (32). A meta-analysis of more than 2,000 cases showed that PYGL was upregulated in several cases, including clear cell renal cell carcinoma, seminoma, and brain cancer, especially in the hypoxic tumor microenvironment (33). In addition, ANXA5 is involved in many physiological and pathological processes, such as cell signal transduction, inflammation, cell growth, and proliferation (34, 35). Abnormally expressed ANXA5 is related to angiogenesis and the progression of glioma (36). ANXA5 can regulate phosphatidylserine (PS)-mediated immunosuppression by binding to PS, increasing the immunogenicity of apoptotic cells, thereby enhancing the immunosuppression of the tumor microenvironment (37). It should be noted that further research is indispensable and in urgent need to validate the roles of these identified vaccine antigens.

To segment the appropriate population for optimal mRNA vaccination, glioma patients were subdivided into three immune subtypes based on immune gene expression profiles. We observed distinct molecular, cellular, and clinical characteristics among the three immune subtypes IS1–3. Patients with IS2 and IS3 tumors prolonged survival in comparison with IS1 in CGGA and TCGA cohorts simultaneously, suggesting that immunotype can be a prognostic biomarker for glioma. We observed that patients overexpressing four antigens ANXA5, FKBP10, MSN, and PYGL showed significantly poorer OS than the low expressed group in IS1–IS3 subtypes. Particularly, the OS prognosis of IS1 was shorter than the other subtypes for these four antigens, indicating that four antigens would be more accurate to serve as vaccines for IS2 and IS3 patients.

Additionally, the immune subtype differed in the expressions of ICD modulators and ICPs. Patients with upregulation of ICD modulators were targetable for mRNA vaccine. Glioma was categorized as C1–C6 except C2 subtypes based on the comprehensive immunotyping research across 33 cancer types (27). In this case, the proportion of five categories varied substantially on IS1–IS3 subtypes. We found C6 associated with the inferior prognoses only clustered into IS1, in agreement with the superior prognosis of IS2 and IS3, and worse survival probability of IS1.

Limited by tumor heterogeneity, complex tumor immune microenvironment (TME), and poor immune access, mRNA vaccine faces resistance in being applied for glioma patients (38, 39). Unsupervised hierarchical clustering of immune-related genes was analyzed rather than conventional supervised learning for the risk prediction model. Defined three subtypes might reflect the different underlying mechanisms regulating tumor immune escape, allowing targeted treatment strategies. The immune desert IS2 and IS3 might be associated with the absence of tumor antigen and antigen-presenting cells, resulting in T cell anergy. Consequently, the use of mRNA vaccine therapy can induce immune infiltration to reinvigorate the immune system in these patients. On the contrary, the IS1 showed an opposite immunologic feature with an immune-hot phenotype hallmarked by an increased immune cell infiltration consequently representing an extremely inflamed microenvironment. Commonly found in the tumor micro-environment, inflammation contributes to immuno-suppression and tumorigenesis, and cancer progression (40, 41). The inflammatory phenotype with higher infiltration of macrophages may partially generate the bad prognosis of patients in IS1 than those in IS2 and IS3.

The immune landscape of glioma indicates considerable heterogeneity between individual patients as well as within the same immune subtype, which narrows down the immune components for developing patient-specific mRNA vaccine treatment. The graph learning-based dimensionality reduction analysis further revealed the intracluster heterogeneity in IS2. Patients in IS2A carried a better survival than other groups in IS2, suggesting that the IS2A type of patients might have better treatment effect for vaccine. Overall, integrating the results of both immune subtypes and the immune landscape of glioma is necessary, and our immunotyping method is reliable and complements the previous classification.

In the face of a global pandemic, mRNA vaccine has entered the limelight as an innovative and promising platform against a range of indications, including COVID-19 (42). Despite its high efficacy rate in preventing COVID-19 mortality and severe infection, the mRNA vaccine needs to be periodically updated to fight the evolving virus with new variants like D614G (43, 44). According to this study, identifying the promising specific antigen and patients with corresponding immune subtypes applicable for mRNA vaccine treatment may help to improve clinical practice in combating COVID-19.

## Conclusions

Four antigens ANXA5, FKBP10, MSN, and PYGL were promising to develop mRNA vaccine against glioma. Patients with subtypes IS2 and IS3 were suitable candidates. Our findings provided valuable stratification for developing antiglioma mRNA vaccines.

## Data Availability Statement

The original contributions presented in the study are included in the article/**Supplementary Material**. Further inquiries can be directed to the corresponding authors.

## Author Contributions

HZ and SL made contribution to design the study, analyzed the data, and drew the figures. HZ, SL, and FC drafted the manuscript. ZP, YL, and GY helped in the substantial revisions of the manuscript. FT and SX contributed to literature search. YZ and JZ helped with data collection and interpretation. CW, GY, and Z-QL supervised the study. FC, JZ, CW, and Z-QL provided the project funding. HZ, SL, and FC contributed equally to this work. All authors read and approved the final manuscript.

## Funding

This study was financially supported by the National Natural Science Foundation of China (81660421, 81860450, and 81660512), Basic Research Project in Guizhou Province (No. (2018) 1425, No. (2021) 467), Young talents Found of Zunyi medical University (18zy-005), Science and Technology in Zunyi (2020-105). National Health Commission of China (2018ZX-07S-011), Medical Science Advancement Program of Wuhan University (TFJC2018003), and Translational Medicine Research Joint Fund of Zhongnan Hospital of Wuhan 1548 University (No. ZNLH201901, ZLYNXM202011).

## Conflict of Interest

The authors declare that the research was conducted in the absence of any commercial or financial relationships that could be construed as a potential conflict of interest.

## Publisher’s Note

All claims expressed in this article are solely those of the authors and do not necessarily represent those of their affiliated organizations, or those of the publisher, the editors and the reviewers. Any product that may be evaluated in this article, or claim that may be made by its manufacturer, is not guaranteed or endorsed by the publisher.

## Supplementary Material

Supplementary Table 1 Identification of immune hub genes of glioma. Forest maps of single factor survival analysis of 10 modules of glioma **(A)**. Bubble plots showing top KEGG terms enriched in the red **(B)** and top GO terms enriched in pink **(C)** mod.

Supplementary Figure 1 Kaplan-Meier OS curves comparing the groups with high and low expressions of ANXA5, FKBP10, MSN, and PYGL in WHO II-IV grade glioma respectively.

Supplementary Figure 2 Kaplan-Meier OS curves comparing the groups with high and low expressions of ANXA5, FKBP10, MSN, and PYGL in IS1-IS3 subype glioma respectively.

Supplementary Figure 3 Distribution of WHO II-IV across IS1- IS3 glioma subtype in CGGA and TCGA cohorts.

## Abbreviations

APCs, antigen-presenting cells; TCGA, The Cancer Genome Atlas; CGGA, Chinese Glioma Genome Atlas; GBM, glioblastoma; LGG, low-grade glioma; FPKM, fragments per kilobase per million; WGCNA, weighted correlation network analysis; GEPIA, gene expression profiling interactive analysis; ANOVA, analysis of variance; ImmPort, Immunology Database and Analysis Portal; GO, Gene Ontology; KEGG, Kyoto Encyclopedia of Genes and Genomes; OS, overall survival; DFS, disease-free survival; PAM, partition around medoids; TIICs, tumor-infiltrating immune cells; DDRTree, discriminative dimensionality reduction with trees; IS, immune subtype; TMB, tumor mutational burden; ICPs, immune checkpoints; ICD, immunogenic cell death; MHC, major histocompatibility complex; MEs, module eigengenes; PC, principal component.
