# Supplementary material for: Dissecting Tumor Antigens and Immune Subtypes of Glioma to Develop mRNA Vaccine
Source: Front Immunol. 2021 Aug 27;12:709986. doi: 10.3389/fimmu.2021.709986 (PMC8429949; doi:10.3389/fimmu.2021.709986)
Supplement: Supplementary Figure 1 — Kaplan-Meier OS curves comparing the groups with high and low expressions of ANXA5, FKBP10, MSN, and PYGL in WHO II-IV grade glioma respectively. [file Image_1.pdf]

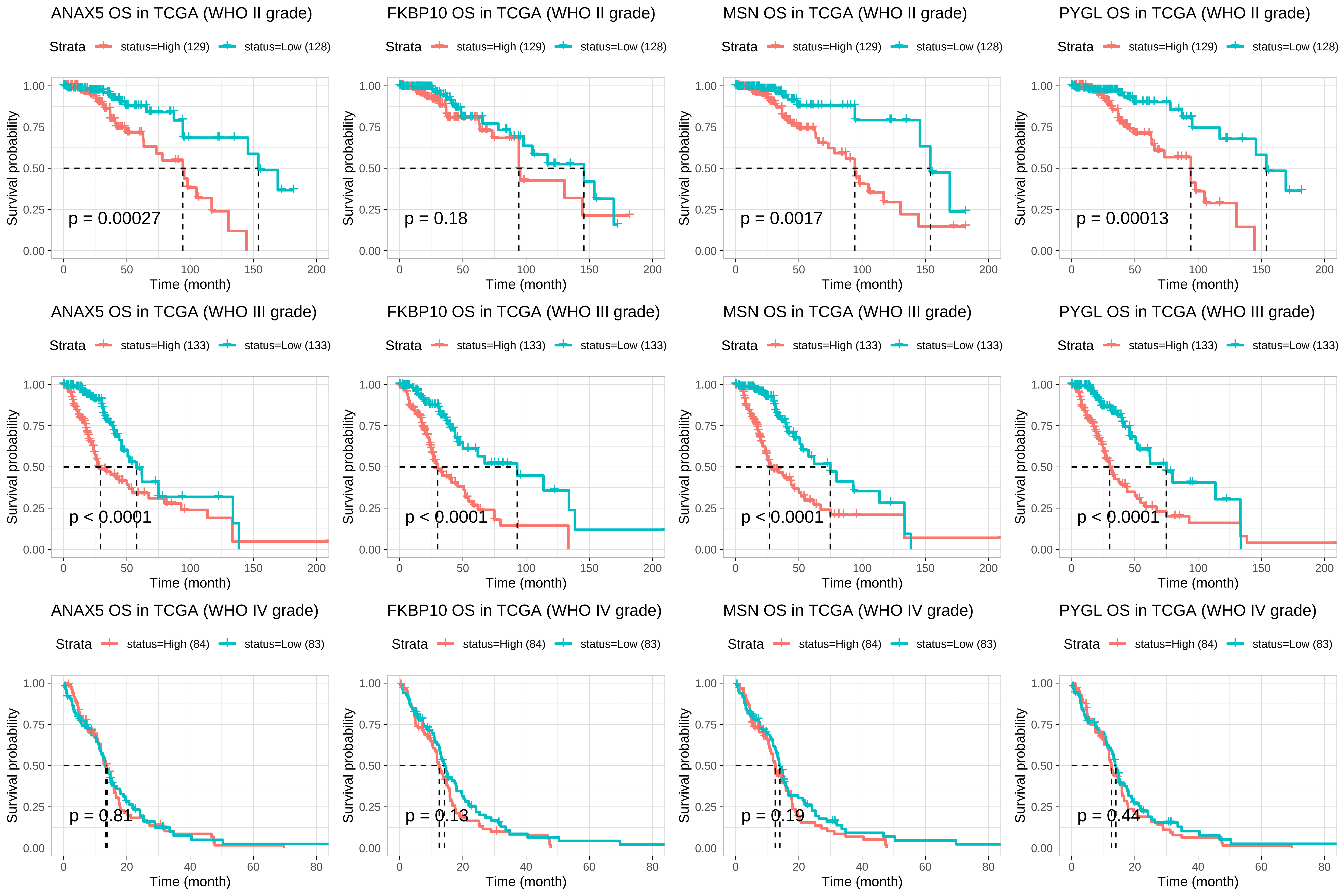
ANAX5 OS in TCGA (WHO II grade)
Strata status=High (129) status=Low (128)
p = 0.00027
FKBP10 OS in TCGA (WHO II grade)
Strata status=High (129) status=Low (128)
p = 0.18
MSN OS in TCGA (WHO II grade)
Strata status=High (129) status=Low (128)
p = 0.0017
PYGL OS in TCGA (WHO II grade)
Strata status=High (129) status=Low (128)
p = 0.00013
ANAX5 OS in TCGA (WHO III grade)
Strata status=High (133) status=Low (133)
p < 0.0001
FKBP10 OS in TCGA (WHO III grade)
Strata status=High (133) status=Low (133)
p < 0.0001
MSN OS in TCGA (WHO III grade)
Strata status=High (133) status=Low (133)
p < 0.0001
PYGL OS in TCGA (WHO III grade)
Strata status=High (133) status=Low (133)
p < 0.0001
ANAX5 OS in TCGA (WHO IV grade)
Strata status=High (84) status=Low (83)
p = 0.81
FKBP10 OS in TCGA (WHO IV grade)
Strata status=High (84) status=Low (83)
p = 0.13
MSN OS in TCGA (WHO IV grade)
Strata status=High (84) status=Low (83)
p = 0.19
PYGL OS in TCGA (WHO IV grade)
Strata status=High (84) status=Low (83)
p = 0.44
Survival probability
Time (month)
