# Supplementary material for: Dissecting Tumor Antigens and Immune Subtypes of Glioma to Develop mRNA Vaccine
Source: Front Immunol. 2021 Aug 27;12:709986. doi: 10.3389/fimmu.2021.709986 (PMC8429949; doi:10.3389/fimmu.2021.709986)
Supplement: Supplementary Figure 2 — Kaplan-Meier OS curves comparing the groups with high and low expressions of ANXA5, FKBP10, MSN, and PYGL in IS1-IS3 subype glioma respectively. [file Image_2.pdf]

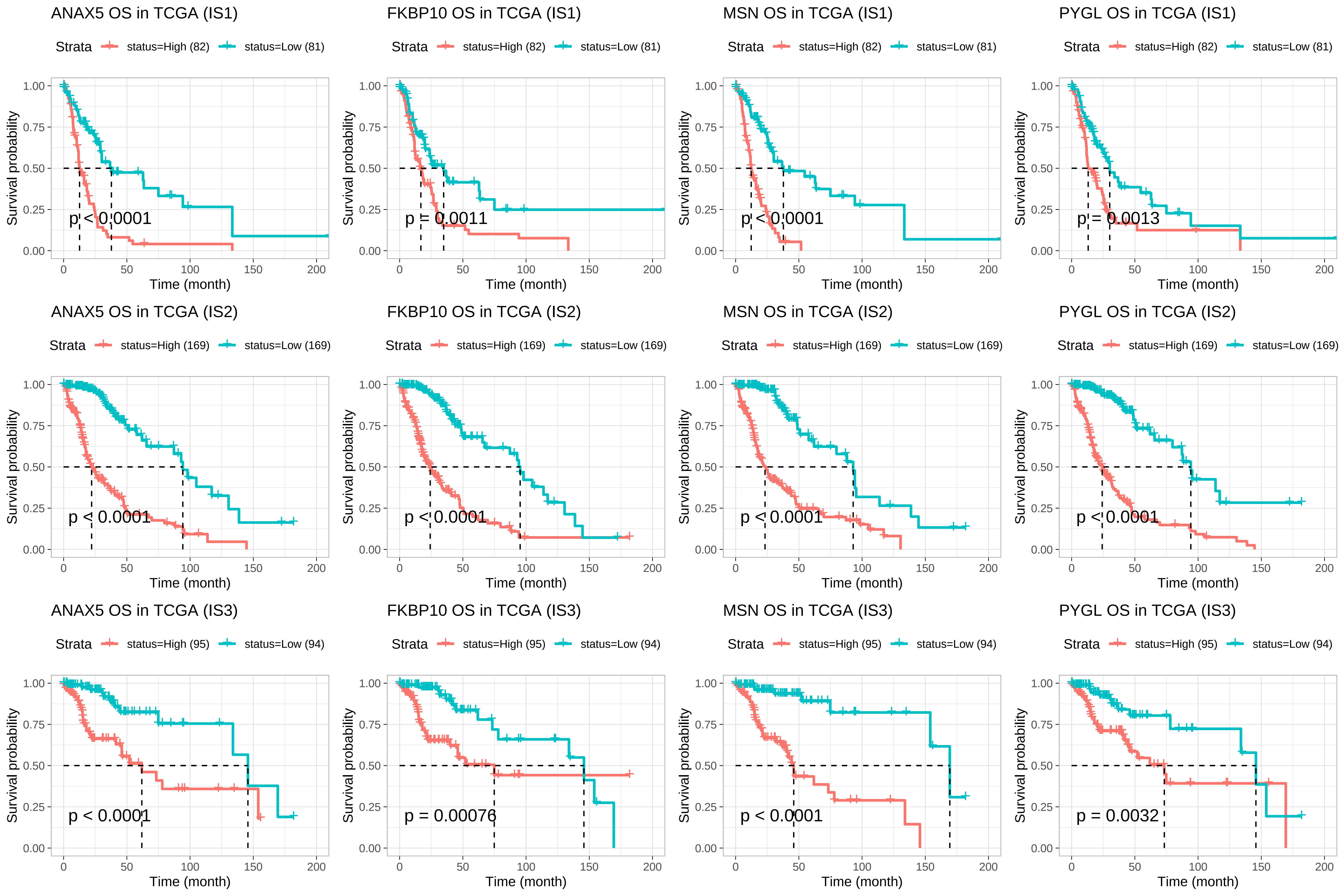

ANAX5 OS in TCGA (IS1)
Strata status=High (82) status=Low (81)
p < 0.0001
FKBP10 OS in TCGA (IS1)
Strata status=High (82) status=Low (81)
p = 0.0011
MSN OS in TCGA (IS1)
Strata status=High (82) status=Low (81)
p < 0.0001
PYGL OS in TCGA (IS1)
Strata status=High (82) status=Low (81)
p = 0.0013
ANAX5 OS in TCGA (IS2)
Strata status=High (169) status=Low (169)
p < 0.0001
FKBP10 OS in TCGA (IS2)
Strata status=High (169) status=Low (169)
p < 0.0001
MSN OS in TCGA (IS2)
Strata status=High (169) status=Low (169)
p < 0.0001
PYGL OS in TCGA (IS2)
Strata status=High (169) status=Low (169)
p < 0.0001
ANAX5 OS in TCGA (IS3)
Strata status=High (95) status=Low (94)
p < 0.0001
FKBP10 OS in TCGA (IS3)
Strata status=High (95) status=Low (94)
p = 0.00076
MSN OS in TCGA (IS3)
Strata status=High (95) status=Low (94)
p < 0.0001
PYGL OS in TCGA (IS3)
Strata status=High (95) status=Low (94)
p = 0.0032
Survival probability
Time (month)
