# Supplementary figures and images for: Dissecting Tumor Antigens and Immune Subtypes of Glioma to Develop mRNA Vaccine
Source: Front Immunol. 2021 Aug 27;12:709986. doi: 10.3389/fimmu.2021.709986 (PMC8429949; doi:10.3389/fimmu.2021.709986)

CGGA

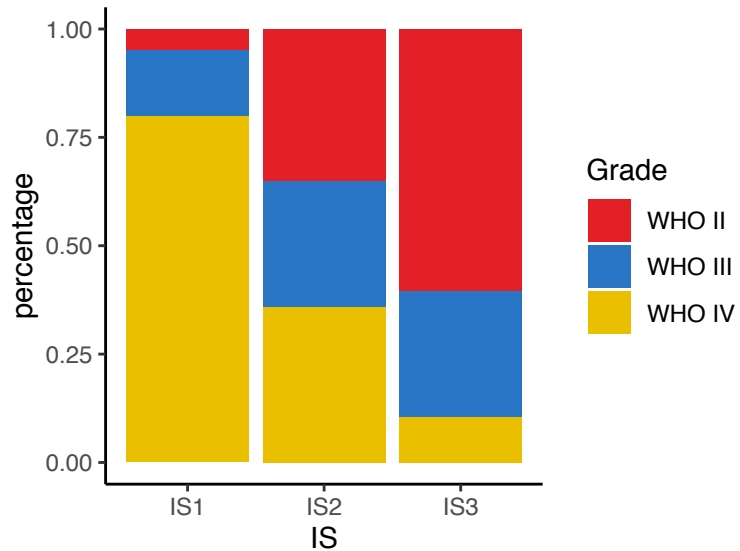

TCGA

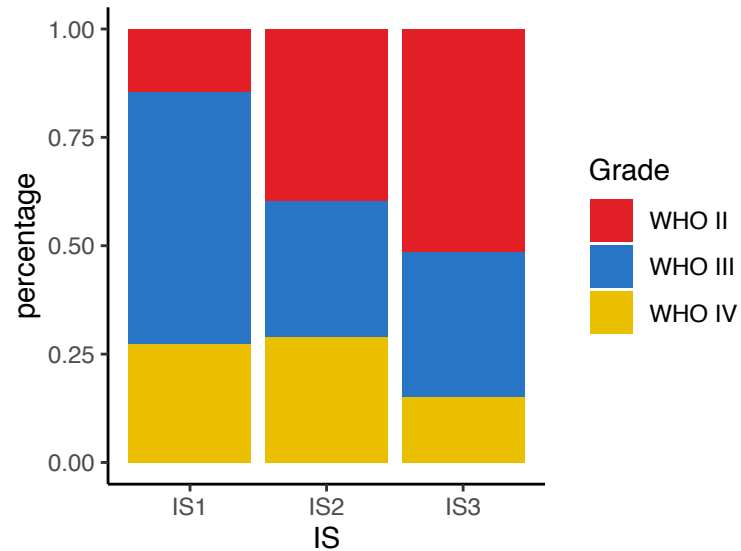

Supplement: Supplementary Figure 3 — Distribution of WHO II-IV across IS1- IS3 glioma subtype in CGGA and TCGA cohorts. [file Image_3.pdf]
